# Phosphorylation of Phylogenetically Conserved Amino Acid Residues Confines HBx within Different Cell Compartments of Human Hepatocarcinoma Cells

**DOI:** 10.3390/molecules26051254

**Published:** 2021-02-26

**Authors:** Cristian Prieto, Juan Montecinos, Gustavo Jiménez, Constanza Riquelme, Daniel Garrido, Sergio Hernández, Alejandra Loyola, Rodrigo A. Villanueva

**Affiliations:** Laboratorio de Epigenética y Cromatina, Fundación Ciencia y Vida, Avda. Zañartu 1482, Ñuñoa, Santiago 7780272, Chile; cr.prietop@gmail.com (C.P.); jmontek@gmail.com (J.M.); gustavo.jimenez.alarcon@gmail.com (G.J.); ccre.escudero@gmail.com (C.R.); daniel.garrido.n@gmail.com (D.G.); zenden21@gmail.com (S.H.)

**Keywords:** hepatitis B virus, HBV, hepatitis B virus X protein, HBx, phosphorylation, subcellular localization, localization regulation

## Abstract

Hepatitis B virus (HBV) is a circular, and partially double-stranded DNA virus. Upon infection, the viral genome is translocated into the cell nucleus, generating the covalently closed circular DNA (cccDNA) intermediate, and forming a mini chromosome. HBV HBx is a small protein displaying multiple roles in HBV-infected cells, and in different subcellular locations. In the nucleus, the HBx protein is required to initiate and maintain viral transcription from the viral mini chromosome. In contrast, HBx also functions in the cytoplasm, where it is able to alter multiple cellular functions such as mitochondria metabolism, apoptosis and signal transduction pathways. It has been reported that in cultured cells, at low expression levels, the HBx protein is localized in the nucleus, whereas at high expression levels, it accumulates in the cytoplasm. This dynamic subcellular distribution of HBx might be essential to exert its multiple roles during viral infection. However, the mechanism that regulates different subcellular localizations of the HBx protein is unknown. We have previously taken a bioinformatics approach to investigate whether HBx might be regulated via post-translational modification, and we have proposed that the multiple nucleocytoplasmic functions of HBx might be regulated by an evolutionarily conserved mechanism via phosphorylation. In the current study, phylogenetically conserved amino acids of HBx with a high potential of phosphorylation were targeted for site-directed mutagenesis. Two conserved serine (Ser25 and Ser41), and one conserved threonine (Thr81) amino acids were replaced by either alanine or aspartic acid residues to simulate an unphosphorylated or phosphorylated state, respectively. Human hepatoma cells were transfected with increasing amounts of the HBx DNA constructs, and the cells were analyzed by fluorescence microscopy. Together, our results show that the nucleocytoplasmic distribution of the HBx protein could be regulated by phosphorylation since some of the modified proteins were mainly confined to distinct subcellular compartments. Remarkably, both HBx Ser41A, and HBx Thr81D proteins were predominantly localized within the nuclear compartment throughout the different expression levels of HBx mutants.

## 1. Introduction

Hepatitis B virus (HBV) is a small, enveloped, hepatotropic virus whose genome is circular, partially double-stranded DNA of about 3.2 kbp, and it is the prototype member of the *Hepadnaviridae* family (genus *Orthohepadnavirus*) [1,2]. Upon infection, the viral genome is translocated to the nucleus of hepatocytes, where it is repaired by the cellular machinery, generating the covalently closed circular DNA (cccDNA) intermediate [3]. This nuclear cccDNA intermediate is then utilized by the cellular transcriptional machinery to generate viral transcripts and genome replication. The cccDNA nuclear intermediate is considered responsible for long-term, persistent infections, since the virus can cause not only acute but chronic hepatitis, cirrhosis, and lead to hepatocellular carcinoma [4]. According to estimations from the World Health Organization, more than 250 million people worldwide are carriers of chronic HBV infections.

**Figure 1:**
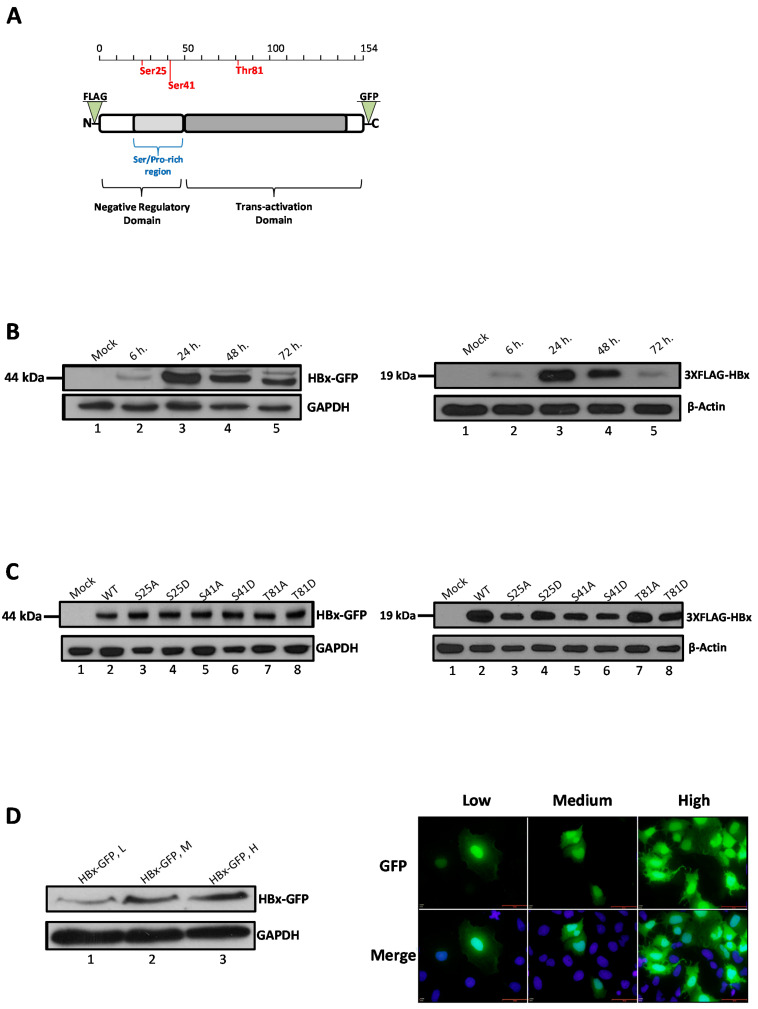
Organization domains of the HBV HBx protein, and expression of the wild-type and point mutant in cells. (**A**) Functional domains of the HBx protein, and point mutated residues. (**B**) Time-course expression of the tagged-HBx protein in human hepatoma cells. Left panel, HBx-GFP construct and right panel, 3xFLAG-HBx protein. (**C**) Expression of HBx point mutant constructs in human hepatoma cells. Left panel, WT HBx-GFP construct and right panel, 3xFLAG-HBx (WT) protein. (**D**) DNA concentration-dependent control expression of the HBx-GFP construct (left panel), and fluorescence of GFP protein in hepatoma cells (right panel). Bar for scale of 50 µm.

The HBx protein is the smallest encoded gene of the virus and is very well conserved among the different viral genotypes. The protein is common in all mammalian members of the viral family but is missing in avian viruses. The primary amino acids sequence of HBx spans 154 residues (predicted about 17 kDa) and is structured into two functional domains (Figure 1A) [5]. The N-terminal first third displays a negative regulatory domain, while the two-thirds C-terminal region has a function as a transactivation region. The negative regulatory domain has been located to the first fifty amino acid residues including a Ser/Pro-rich (residues 21–50) dimerization region that is required for HBx dimerization. The N-terminal region (residues 1–50) of HBx has been shown to be important for cellular transformation. The transactivation domain has been mapped to the region between residues 53 and 142 and the negative regulatory domain is dispensable for this function, and actually represses HBx transactivation [6,7,8,9].

The tumorigenic potential of HBV has been mainly assigned to the expression of the viral HBx protein, which displays multiple cellular roles, possibly leading to hepatocarcinogenesis. The HBx protein has been found to be overexpressed in samples of human hepatocellular carcinoma (HCC), and its overexpression in hepatocyte cultures and transgenic mice has resulted in an oncogenic phenotype in some study models [10,11]. Moreover, a variety of different cytoplasmic signal transduction cascades appear to be altered by HBx including the extracellular signal-regulated kinase (ERK), Janus kinase/STAT (JAK/STAT), adhesion kinase (FAK), proline-rich tyrosine kinase 2 (Pyk2), stress-activated protein kinases/NH2-terminal-Jun kinase (SAPK/JNK), Ras-Raf-mitogen-activated protein kinase (Ras-Raf-MAPK), protein kinase B (PKB/Akt), and Wnt/β-catenin signaling pathway [12,13]. Conversely, nuclear HBx has been shown to transactivate and upregulate the expression of both viral and host gene promoters [14]. This ability of HBx to transactivate the transcription of viral/host genes is thought to go through indirect interaction with general transcription factors such as TFIIH, TFIIB, and RBP5, a subunit of mammalian RNA polymerase [6]. It was recently shown that HBx could interact and cooperate with CREB-binding protein (CBP)/p300 to synergistically enhance CREB activity [15]. In contrast, multiple pathways of DNA repair have also been found to be affected by the expression of HBx. Moreover, it was found that both HBx and telomerase were highly expressed in hepatoma and liver cirrhosis tissues, and that HBx could upregulate the expression and activity of the catalytic subunit of the telomerase (hTERT) [16]. Together, these findings suggest that HBx expression may play a role in the development of HCC by targeting different cellular pathways.

Subcellular localization of the HBx protein has been a matter of controversial debate for decades. Since little is known about its regulatory mechanism and, although interactions with several cytoplasmic and/or nuclear proteins have been shown in in vitro assays, there is no clear consensus as to where HBx is preferentially located in infected hepatocytes. Several in vitro studies have reported that the location of HBx is preferably in the cytoplasm [17,18,19,20], while other groups have reported that it is localized to the nucleus [21]. Recently, using a novel monoclonal antibody and HBV-infected primary human hepatocytes, it was shown that HBx was mainly located in the nucleus [22]. As a possible explanation for these apparently contradictory results from different laboratories, it has been observed that when HBx is expressed at very low levels, it is predominantly localized in the nucleus; when the protein is expressed at higher levels, it accumulates in the cytoplasm [23,24]. Whereas, when HBx is expressed at intermediate levels of expression, it localizes to both nucleus and cytoplasm compartments. This information seems to indicate that the subcellular localization of the protein is influenced by its relative abundance. However, subcellular distribution studies of HBx in human liver biopsies using an anti-HBx rabbit polyclonal antiserum have shown dual location, where HBx is preferentially localized in the cytoplasm, although a significant number of cells showed nuclear localization [25].

Given the different roles that HBx has shown in several intracellular locations and the multiple interactions with distinct host cell factors, it has been difficult to elucidate a mechanism to explain the regulation of its functions. Since the primary sequence of HBx shows no obvious organellar targeting signal, a functional alternative could be that the HBx protein is regulated by post-translational modification. Indeed, several groups have been able to determine that HBx becomes phosphorylated when expressed in insect cells and human hepatoma cells HepG2 [26,27]. Moreover, the recombinant HBx was found to be in vitro phosphorylated by both protein kinase C (PKC) and mitogen protein kinase (MAPK). Phospho-amino acid analysis showed that the phosphorylated residues of the protein could correspond to Ser residues [28]. These studies were later confirmed, determining that Ser residues at position 31 and 41 are phosphorylated by protein kinases Akt 1 and ERK1/2, respectively [29,30]. Moreover, peptidyl propyl isomerase (peptidyl-prolyl cis-trans isomerase NIMA-interacting 1, Pin1), often overexpressed in HCC, is able to interact with HBx when the Ser 41 residue is phosphorylated [31]. Finally, applying bioinformatic tools to investigate if the HBx protein might be regulated via phosphorylation, our group previously found that the phylogenetically conserved residues Ser25 and Ser41 (both within the negative regulatory domain), and Thr81 (in the transactivation domain) are highly predicted to be phosphorylated [32]. In the current study, to validate our prediction data, phylogenetically conserved amino acids of HBx with a high potential of phosphorylation were targeted for site-directed mutagenesis, and replaced by either alanine or aspartic acid residues to simulate an unphosphorylated or phosphorylated state, respectively. Human hepatoma cells were transfected with increasing amounts of HBx DNA constructs, and the cells were analyzed by indirect immunofluorescence and epifluorescence microscopy. Together, our results showed that the nucleocytoplasmic distribution of the HBx protein could be regulated by phosphorylation since some of the modified proteins were mainly confined to different subcellular compartments.

## 2. Results

We have recently taken a bioinformatics approach to investigate whether the viral protein HBx might be regulated via phosphorylation by an evolutionarily conserved mechanism. We found that phylogenetically conserved residues Ser25 and Ser41 both located in the negative regulatory domain of the primary sequence of HBx, and Thr81, within the trans-activation domain, displayed a high potential to become phosphorylated (Figure 1A). In the mentioned study, we proposed that the different roles of HBx displayed in different subcellular locations might be regulated by an evolutionarily conserved mechanism of post-translational modification via phosphorylation [32]. Additionally, our laboratory has recently described the cloning and full sequence of a functional HBV isolate (named HBV 4.5) recovered from samples of a Chilean infected chronic patient (GenBank KM233681) [33], and we also reported its replication in tissue cultured cells [34]. To perform studies to express and examine the HBx protein, we amplified and subcloned the coding sequence of the protein into a cloning vector. To test if Ser25, Ser41, or Thr81 were involved in phosphorylation regulation of HBx, we performed site-directed mutagenesis on these phylogenetically conserved residues (Tables S1 and S2).

Thus, we generated the point mutants S25A, S41A, and T81A, which all represent non-phosphorylatable residues, and S25D, S41D, and T81D, which all represent phosphorylated residues as a surrogate approach to investigate the role of these positions in regulation. We then moved the point mutated HBx DNA fragments to two different expression vectors: pcDNA3.1-3xFLAG or pAcGFP1-N1. For the case of wild-type and point mutants in the expression vector pcDNA3.1-3xFLAG, the viral HBx proteins were expressed as a fusion polypeptide containing the 3xFLAG tag at the N-terminus of HBx, whereas in the wild-type and point mutants in the expression vector pAcGFP1-N1, the viral HBx proteins were expressed as a fusion polypeptide containing the GFP tag reporter protein at the C-terminus of HBx.

HBx protein constructs were first tested to determine the peak of abundance after transfection in a time-course experiment. HBx DNA transfections were performed, and cells were analyzed after 6-, 24-, 48-, or 72-h for protein expression by western blot. As shown in Figure 1B, the peak of abundance of both HBx-GFP (HBx fused to the Green Fluorescent Protein), and 3xFLAG-HBx were at a similar time-point as the major expression was 24-h post-transfection (Figure 1B, left and right panel, lanes 3). Thus, having determined that 24-h post-transfection was the peak abundance of protein expression for the two WT HBx constructs, we tested the expression of the different HBx point mutant constructs under these conditions. Cells were transfected, and protein expression after 24-h was assayed by western blot. Figure 1C left and right panels show the expression profiles of HBx-GFP and 3x-FLAG-HBx (lanes 2), and constructs (lanes 3–8), respectively.

These figures show that at 24-h post-transfection, all HBx point mutant constructs were abundantly expressed, and all were in a similar proportion. Therefore, 24-h post-transfection was the optimal time-point to analyze the behavior of the proteins in the following experiments. Conversely, in previous works, it was shown that different expression levels of HBx determine its different subcellular distributions. Thus, depending on the level of expression, HBx first localizes to the nucleus at low expression levels, and then at higher expression levels, it localizes to the cytoplasm [23,24]. For these reasons, we tested different amounts of DNA to achieve different levels of HBx abundance. As control experiments, we first tested the expression of the HBx-GFP construct as well as GFP expression (empty vector of pAcGFP1-N1) using three different amounts of input DNA: low (L), medium (M), and high (H). Cells were transfected with either the HBx-GFP construct or GFP empty vector, and cells were processed either for western blot (HBx-GFP) or fluorescence microscopy (GFP alone). Results are shown in Figure 1D. The left panel shows that HBx-GFP displayed a level of expression directly related with the amount of DNA input transfected, thus, the more transfected DNA, the more HBx expression. The right panel shows the fluorescence microscopy of the GFP reporter protein. The figure shows that the GFP signal was homogeneously distributed over both the nucleus and cytoplasm of transfected cells. This localization pattern was observed under conditions of low, medium, and high amounts of transfected DNA. However, the number of transfected cells was also affected by the amount of transfected DNA, as a higher number of cells were transfected with more input DNA with a general transfection efficiency of about 50%.

**Figure 2:**
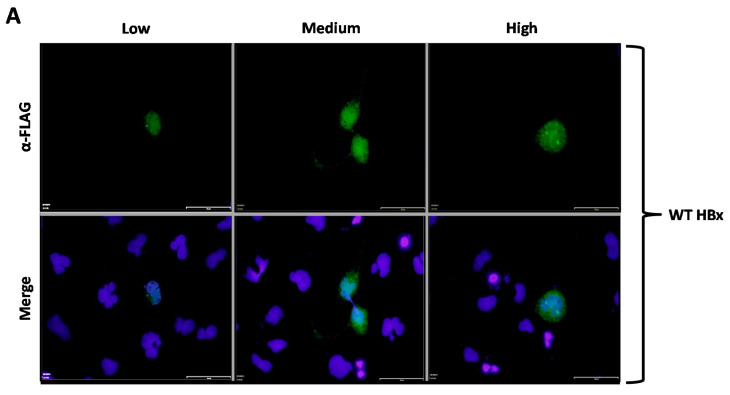

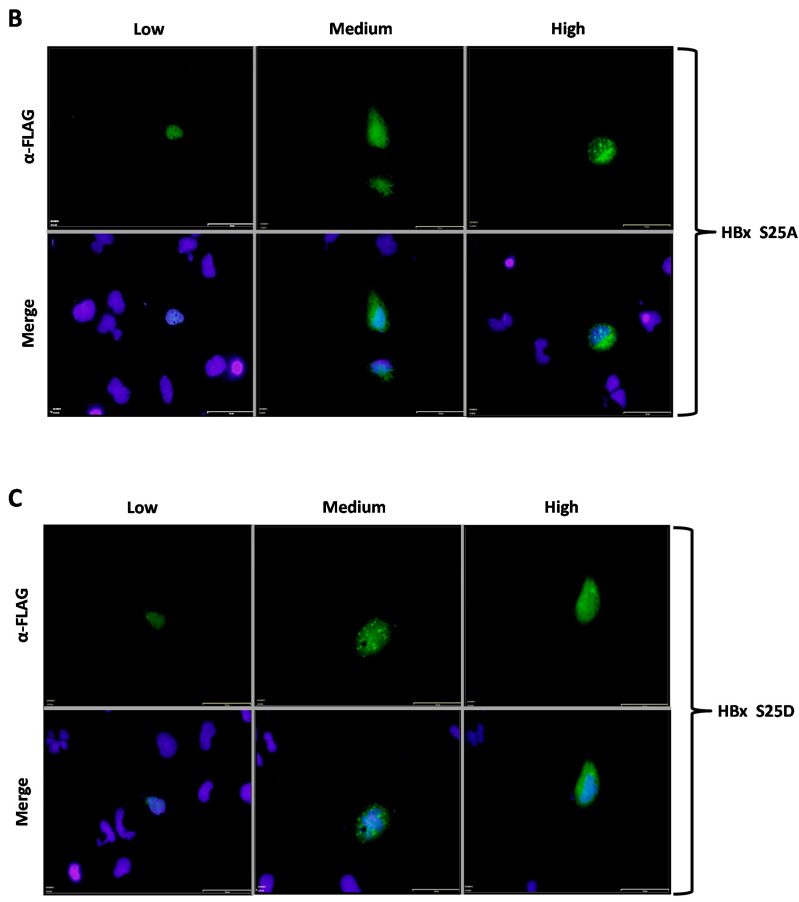

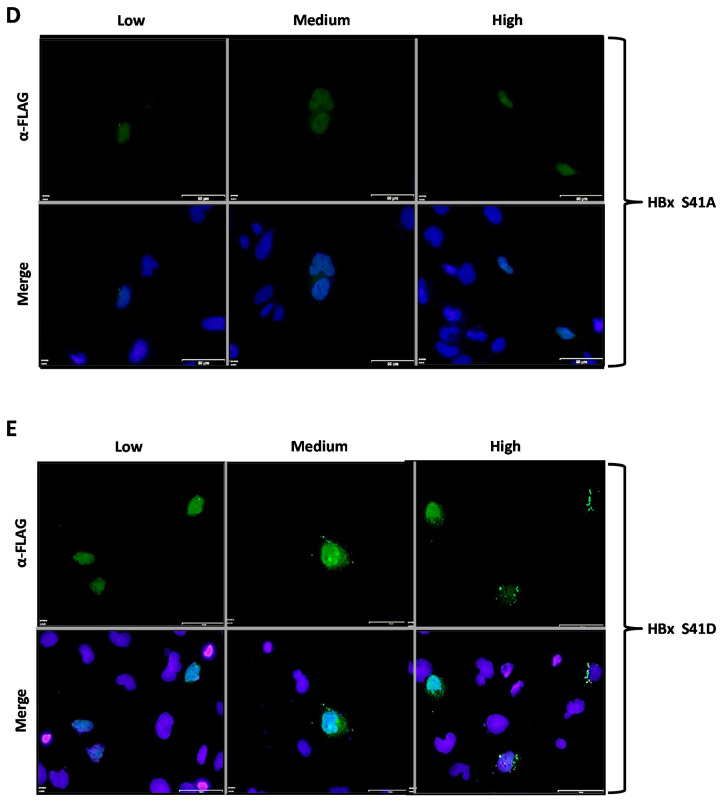

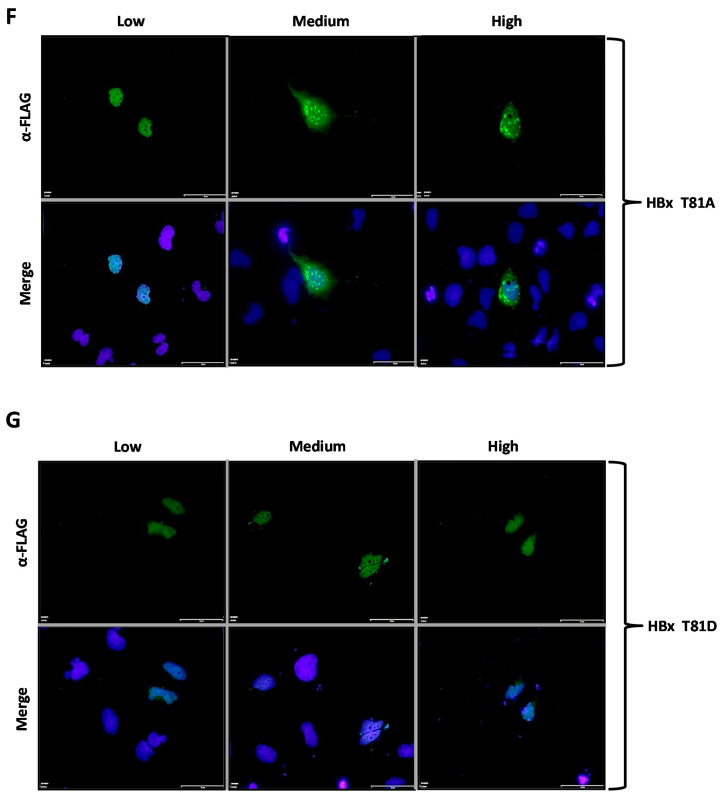
Immunofluorescence of the FLAG-tagged HBx protein and point mutant in cells. (**A**) Immunofluorescence of hepatoma cells transfected with low (left), medium (middle), and high (right) amounts of plasmid DNA containing WT 3xFLAG-HBx. Bottom panel shows nuclear DAPI signal merged with that of α-FLAG. (**B**) 3xFLAG HBx S25A, (**C**) 3xFLAG HBx S25D, (**D**) 3xFLAG HBx S41A, (**E**) 3xFLAG HBx S41D, (**F**) 3xFLAG HBx T81A, (**G**) 3xFLAG HBx T81D. Bar for scale of 50 µm.

We then analyzed how the subcellular localization of HBx was affected depending on the phosphorylation state of the conserved residues Ser25, Ser41, or Thr81. Our first approach was to use indirect immunofluorescence associated with the 3xFLAG-HBx construct and point mutants using a commercially available monoclonal antibody raised against the FLAG tag. For this aim, cells were transfected with the 3xFLAG-HBx WT construct or point mutants, and after 24-h, coverslips were processed for immunofluorescence using a primary α-FLAG monoclonal antibody, and a secondary antibody conjugated to Alexa 488. Samples were observed under fluorescence microscopy. Positive cell images were magnified to facilitate their examination. Results are shown in Figure 2A–G. As shown in Figure 2A, transfection of a low amount of the WT 3xFLAG-HBx DNA construct resulted in the detection of an intense signal coming from the nucleus (Figure 2A, Low, left panel), whose signal seemed to completely merge that of nuclear DNA by DAPI. An increasing amount of transfected DNA (Figure 2A, Medium, middle panel) resulted in combined nucleocytoplasmic staining, where the signal began to appear from the cytoplasm, and the staining had a punctuated distribution near the perinuclear region [23]. Further increases in the amount of the transfected DNA for the WT 3x-FLAG-HBx construct (Figure 2A, High, right panel) showed the staining signal came mainly from the whole cytoplasm. Thus, consistent with previous studies from different laboratories, at low amounts of transfected DNA, the WT HBx protein localized mainly to the nucleus, whereas increasing amounts of transfected DNA turned the WT protein localization toward the cell cytoplasm [23,24]. We then analyzed the behavior of both HBx S25A and S25D point mutants. Results for these mutants are shown in Figure 2B,C, respectively. As for the WT HBx protein, transfection of a low quantity of either of these point mutants, resulted in an intense signal coming from the nucleus, whose signal seemed to completely overlap that of nuclear DAPI (nuclear DNA) (Figure 2B,C, Low, left panels). Similar with the WT HBx protein, increasing amounts of transfected DNA of either of these mutants (Figure 2B,C, Medium and High, middle and right panels, respectively) moved the staining mainly to the cell cytoplasm. Together, these results indicated that both the HBx S25A and S25D point mutants did not show significant differences in their behaviors with respect to that of WT HBx. In contrast, Figure 2D,E showed the subcellular localization of the HBx S41A and S41D point mutants, respectively. Similar to all previously tested WT HBx and constructs, transfection of a low quantity of plasmid DNA of either HBx S41A and S41D point mutants resulted in an intense signal arising from the nuclei, whose signal seemed to completely overlap that of nuclear DAPI (nuclear DNA) (Figure 2D,E, Low, left panels). Importantly, increasing amounts of transfected DNA of HBx S41A, medium as well as high quantities, maintained the intensity of the signal exclusively from the nuclei (as it mainly merges with DAPI), making a marked difference with the WT HBx protein. Thus, HBx S41A maintained a nuclear localization throughout the range of tested DNA (Figure 2D, Low, Medium and High panels). In contrast, HBx S41D behaved as the WT HBx protein, where the signal turned to be within the cell cytoplasm (Figure 2D, Medium and High, middle and right panels).

Figure 2F,G show the subcellular localization of the HBx T81A and T81D point mutants, respectively. Similar to the previously tested WT HBx and constructs, transfection of a low quantity of plasmid DNA of either HBx T81A or T81D point mutants resulted in an intense signal arising from the nuclei, whose signal seemed to completely overlap that of the nuclear DAPI (nuclear DNA) (Figure 2F,G, Low, left panels). Increasing amounts of transfected DNA of HBx T81A changed the signal to be within the cell cytoplasm and, thus, this mutant behaved as the WT HBx protein (Figure 2F, Medium and High, middle and right panels). In contrast, the HBx T81D point mutant maintained the intensity of the signal exclusively from the nuclei (as it mainly merges with DAPI) during the range of DNA amount transfected (Figure 2G, Medium and High, middle and right panels).

Thus, this first set of experiments identified that some HBx point mutants behaved as the WT HBx protein. Conversely, we identified two point mutants HBx S41A, and HBx T81D that localized to the nucleus throughout the range of protein expression levels tested.

In the next set of experiments, we analyzed the effect of phosphorylation on the HBx protein fused to the N-terminus of the reporter protein GFP. Studies of subcellular localization of proteins fused to GFP displayed several advantages over other techniques such as a high sensitivity, low noise-to-signal ratio, and a much better resolution of the detected signal [35].

**Figure 3:**
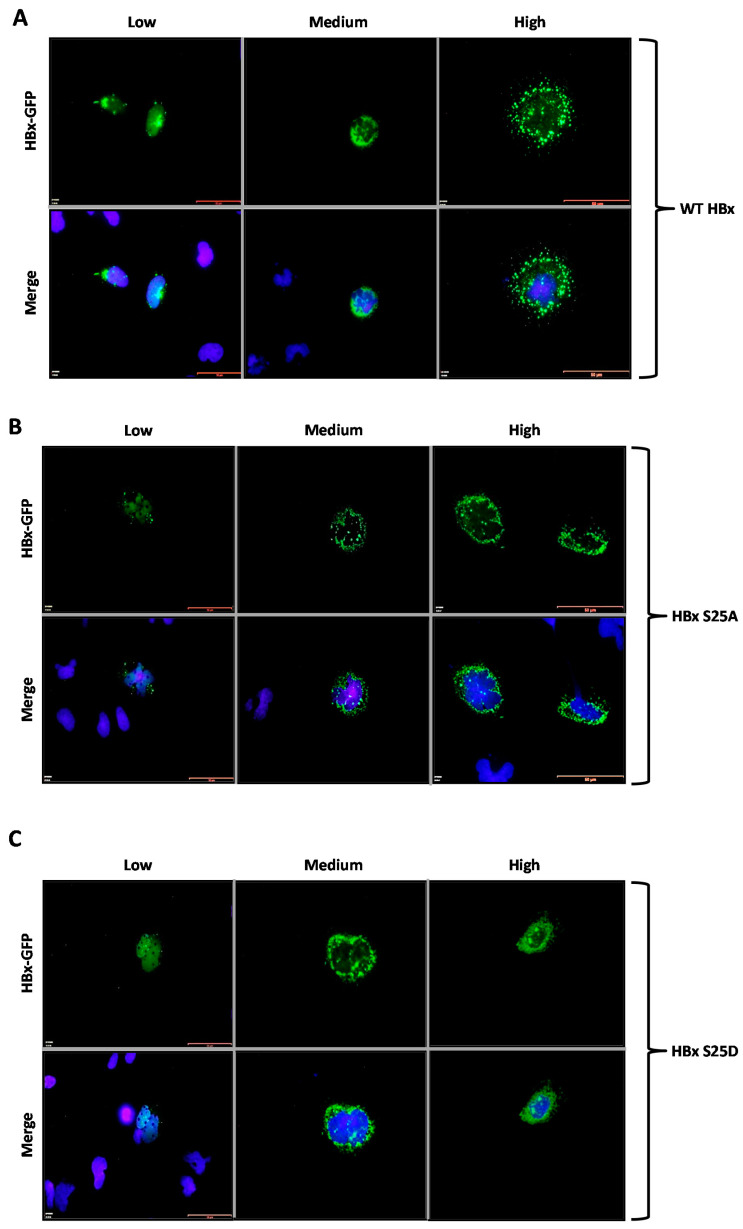

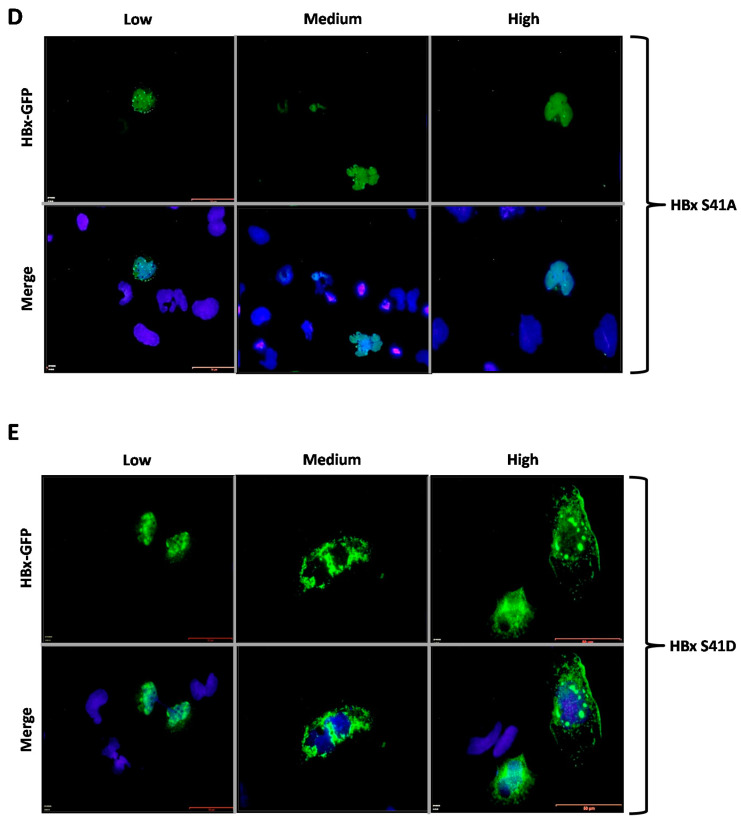

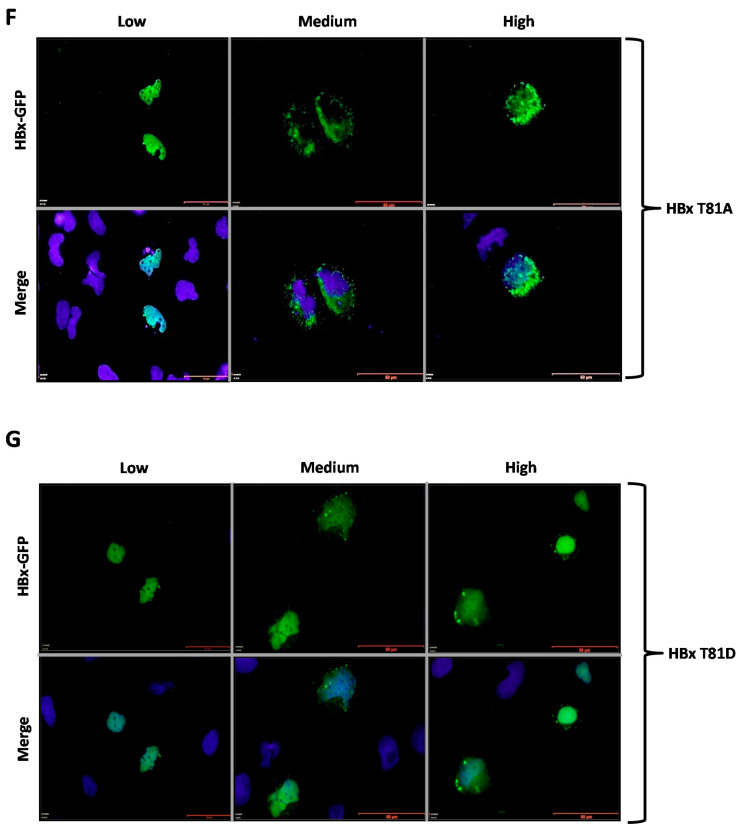
Fluorescence of the HBx-GFP protein and point mutant in cells. (**A**) Fluorescence of hepatoma cells transfected with low (left), medium (middle), and high (right) amounts of plasmid DNA containing HBx protein tagged with GFP. Bottom panel shows nuclear DAPI signal merged with that of GFP fluorescence. (**B**) HBx S25A, (**C**) HBx S25D, (**D**) HBx S41A, (**E**) HBx S41D, (**F**) HBx T81A, (**G**) HBx T81D. Bar for scale of 50 µm.

Cells were transfected with WT HBx-GFP and point mutants, and after 24-h, the coverslips were processed for fluorescence microscopy. Results are shown in Figure 3A–G. As shown in Figure 3A, transfection of a low amount of the WT HBx-GFP DNA construct resulted in the detection of a very intense signal coming from the nuclei, whose signal seemed to completely merge with that of the nuclear DNA by DAPI. Since transfection of cells with the empty vector (for expression of GFP protein alone) had previously shown a uniform distribution throughout both nuclei and cytoplasm (Figure 1D, right panel), these results indicated that the behavior of HBx was not affected by GFP localization. In contrast, an increasing amount of transfected DNA (Figure 3A, Medium, middle panel) resulted in combined nucleocytoplasmic staining, where the signal began to appear from the cytoplasm, and the staining had a punctuated distribution near the perinuclear region 23. Further increases in the amount of transfected DNA for the WT HBx-GFP construct (Figure 3A, High, right panel) showed the staining arose mainly from the cytoplasm, showing a granular, punctuated distribution of the protein, similar to that observed in immunofluorescence experiments (Figure 2A), although much more intense.

To quantitatively uncover all forms of HBx localization, we also carried out a meticulous analysis of HBx subcellular distribution. Cells were transfected with one fixed amount of DNA (high amount) for WT HBx-GFP as well as point mutants, and after 24-h, coverslips were processed for fluorescence. In total, 200 positive cells were analyzed in three independent experiments, and the expression of HBx-GFP proteins was carefully associated with the cytoplasm, nucleus, or nucleocytoplasmic compartments, with respect to DAPI-positive nuclear staining. Data are presented in Figure 5. In the case of WT HBx-GFP, the protein localized up to in 41% in the cell cytoplasm, 23% was nucleocytoplasmic, and 35% was a nuclear protein. Overall, these results indicated that under our experimental conditions, at a high level of expression, the HBx protein not only localized in the cytoplasm, but also within the cell nucleus. These data were more consistent with a WT HBx protein functional in both the nucleus and cytoplasm within the same cell, and with previously analyzed human liver biopsy profiles [25].

We then analyzed the behavior of both point mutants HBx S25A-GFP and HBx S25D-GFP proteins. Results for these mutants are shown in Figure 3B,C. As for the WT HBx protein, transfection of a low quantity of either of these point mutants resulted in an intense signal arising from the nuclei, whose signal completely overlapped that of the nuclear DAPI (nuclear DNA) (Figure 3B,C, Low, left panel). Similar to the WT HBx protein, increasing amounts of transfected DNA of either of these mutants (Figure 3B, Medium and High, middle and right panels) moved the staining toward the cell cytoplasm. Together, these results indicated that both HBx S25A-GFP and HBx S25D-GFP did not show significant differences in their subcellular localization with respect to that of WT HBx. As shown in Figure 5, quantification of the subcellular distribution of these mutants was similar to that of WT HBx where HBx S25A was 38% distributed in the cytoplasm, 26% of the cases displayed a nucleocytoplasmic distribution, and 35% was nuclear, whereas for HBx S25D, the data were 42% cytoplasmic, 20% nucleocytoplasmic, and 37% nuclear protein.

Figure 3D,E show the subcellular localization of the HBx S41A-GFP and S41D-GFP point mutants. Similar to the previously tested WT HBx and constructs, transfection of a low quantity of plasmid DNA of either HBx S41A or S41D point mutants resulted in an intense signal coming mainly from the nuclei, whose signal completely overlapped that of the nuclear DAPI (nuclear DNA) (Figure 3D,E, Low, left panels). Importantly, increasing amounts of transfected DNA of HBx S41A-GFP maintained the intensity of the signal exclusively from nuclei (as it mainly merged with DAPI), whereas, HBx S41D-GFP behaved as the WT HBx protein where the signal was within the cell cytoplasm (Figure 3D,E, Medium and High, middle and right panels). For quantitative results of these mutants, as shown in Figure 5, for HBx S41A, there was a significant reduction of the protein at the cytoplasm with 28%, 21% of the protein was nucleocytoplasmic, and there was a significant increase of the protein at the nucleus in 50% of the cases. Therefore, unlike the WT HBx protein, HBx S41A was a predominantly nuclear protein. Whereas for HBx S41D, this mutant was 45% cytoplasmic, 20% nucleocytoplasmic, and 34% nuclear, whose profile was similar to that displayed by the WT protein.

Figure 3F,G show the subcellular localization of the HBx T81A-GFP and HBx T81D-GFP point mutants. Similar to the previously tested WT HBx and constructs, transfection of a low quantity of plasmid DNA of either HBx T81A-GFP or HBx T81D-GFP point mutants resulted in an intense signal coming from the nuclei, whose signal completely overlapped that of nuclear DAPI (nuclear DNA) (Figure 3F,G, Low, left lane). Increasing amounts of transfected DNA of HBx T81A-GFP showed the signal to be within the cell cytoplasm as this mutant behaved as the WT HBx protein, as shown in Figure 5, where the HBx T81A was 41% cytoplasmic, 19% nucleocytoplasmic, and 39% nuclear. In contrast, the HBx T81D-GFP point mutant maintained the intensity of the signal exclusively from the nuclei (as it mainly merged with DAPI) in the range of the transfected DNA (Figure 3G, Medium and High, middle and right panels). Quantitative analyses of mutant HBx T81D indicated that the protein was 31% cytoplasmic, 18% nucleocytoplasmic, and there was a significant increase in the localization of the HBx T81D protein mutant at the nucleus with a value of 50%. Thus, unlike the WT HBx protein, HBx T81D was a predominantly nuclear protein.

**Figure 4:**
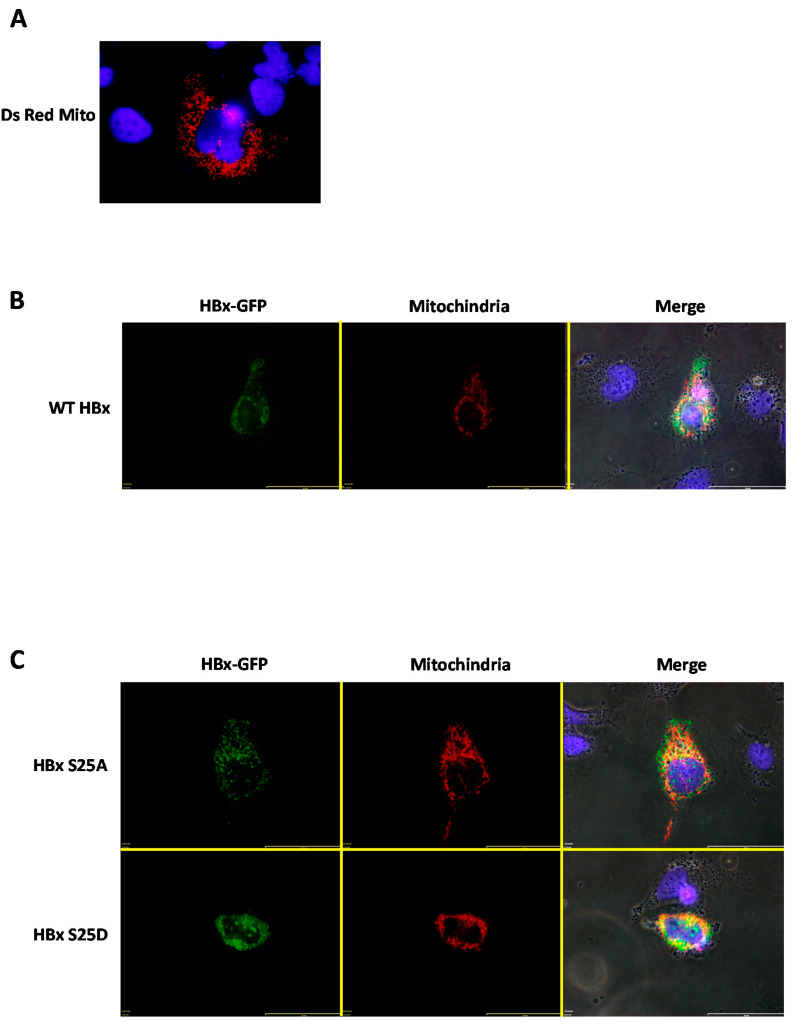

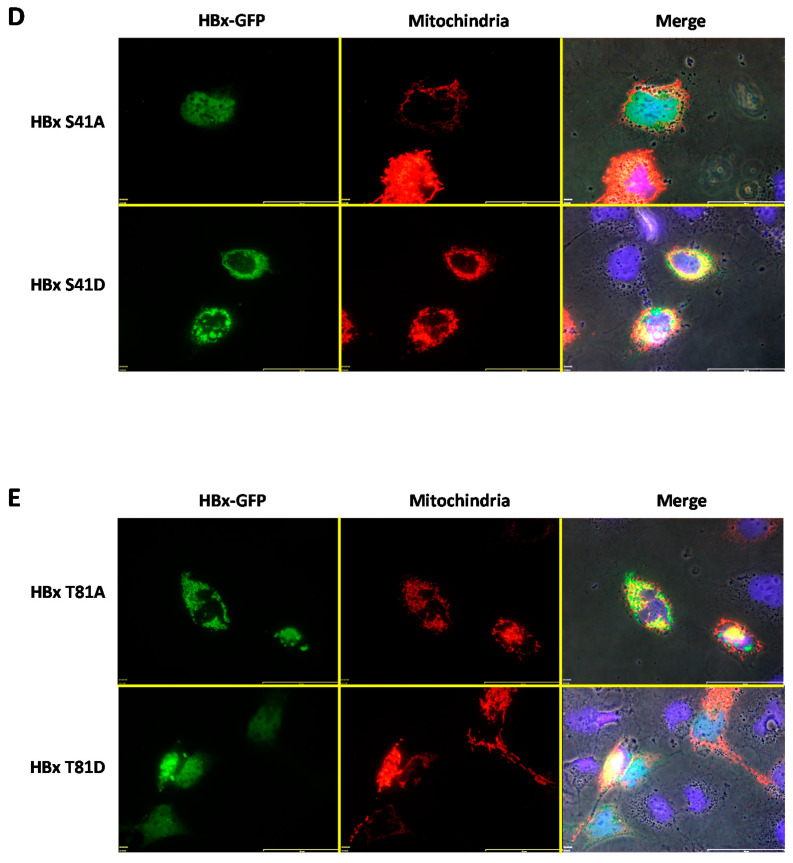
Fluorescence of the HBx-GFP protein and point mutants in cells co-transfected with DsRed2-Mito. (**A**) Fluorescence of hepatoma cells transfected with pDsRed2-Mito. (**B**) Fluorescence of hepatoma cells co-transfected with pDsRed2-Mito and WT HBx-GFP. (**C**) Fluorescence of hepatoma cells co-transfected with pDsRed2-Mito and either HBx S25A-GFP (upper panel) or HBx S25D-GFP (lower panel). (**D**) Fluorescence of hepatoma cells co-transfected with pDsRed2-Mito and either HBx S41A-GFP (upper panel) or HBx S41D-GFP (lower panel). (**E**) Fluorescence of hepatoma cells co-transfected with pDsRed2-Mito and either HBx T81A-GFP (upper panel) or HBx T81D-GFP. Bar for scale of 50 µm.

In previous works, it was determined that a fraction of the HBx protein was capable of associating with the mitochondria, and this association generated mitochondrial aggregation near the perinuclear space [36,37]. For a more detailed analysis of the subcellular location of WT HBx and point mutants with respect to cytoplasmic mitochondria, we carried out co-transfection experiments to overexpress the HBx-GFP protein and point mutants along with a plasmid vector (pDsRed2-Mito) encoding a mitochondrial targeting sequence fused to a red fluorescent protein, DsRed (from *Discosoma* sp) [38,39]. This resulted in an intense red signal as a reporter/marker for subcellular localization of both the cytoplasm and mitochondria. Thus, by transfecting human hepatoma cells with only the mitochondrial targeting vector (pDsRed2-Mito, empty vector), a normal labeling distribution of cytoplasmic mitochondria was observed (Figure 4A).

We then went to co-express WT HBx-GFP together with the pDsRed2-Mito plasmid vector. For this set of experiments, we used a high amount of plasmid DNA, and analyzed the transfected cells after 24-h post-treatment. As shown in Figure 4B, when co-expressing the DsRed2-Mito plasmid vector together with WT HBx-GFP, this image showed that WT HBx-GFP was mainly cytoplasmic. As a result of co-expression with the fusion DsRed2-Mito protein, the mitochondrial distribution was affected, observing cytoplasmic mitochondrial aggregates within the perinuclear region (Figure 4B, Merge panel) [36,37]. Additionally, there was a slight co-localization of the cytoplasmic WT HBx-GFP signal with that of the DsRed2-Mito protein. Consistent with our previous data, both HBx S25A-GFP and HBx S25D-GFP displayed a behavior similar to that of the WT HBx protein (Figure 4C, upper and lower panel, respectively). In contrast, the image showed that co-transfection of DsRed2-Mito with HBx S41A-GFP resulted in the point mutant localized exclusively within the nuclear compartment, and thus did not colocalize with the mitochondrial signal, which arose from the perinuclear area (Figure 4D, upper panel). As observed in the mentioned image, this point mutant displayed a minor effect on the formation of mitochondrial aggregates, with the mitochondria being more distributed throughout the cytoplasm.

After co-transfection of either the HBx S41D-GFP or HBx T81A construct and DsRed2-Mito, these cytoplasmic HBx point mutants showed a slight co-localization with the mitochondrial fusion protein reporter, as shown in Figure 4D (lower panel) and Figure 4E (upper panel), respectively. In contrast, in the case of HBx T81D-GFP, co-transfection with DsRed2-Mito indicated that this point mutant of HBx (at a range amount of transfected DNA) was localized mostly within the nuclear compartment and thus did not colocalize with the mitochondrial signal that arose from the perinuclear area (Figure 4D, upper panel). As observed in the mentioned image, this point mutant displayed a minor effect on the formation of mitochondrial aggregates, with the mitochondria being more regularly distributed throughout the cytoplasm.

## 3. Discussion

Chronic HBV infection is widely related with the development of hepatocellular carcinomas. The HBV protein HBx is an oncogenic factor that can act as a multifunctional regulator to modulate, for example, cell cycle progression, protein degradation, signal transduction pathways, apoptosis, gene transcription, and genetic stability via either direct or indirect interactions with target cell factors [40]. The primary subcellular localization of HBx is nuclear, as shown herein at low levels of expression for all tested WT HBx and point mutant constructs. As the levels of expression of WT HBx protein increased, fractions of the protein were also localized to the cytoplasm. However, for both mutants HBx S41A and HBx T81D, we found that these two proteins were predominantly nuclear proteins. The dynamic subcellular distribution of HBx must be critical for its different functions at different stages during the HBV life cycle. The trans activational roles of HBx may be carried out either in the cytoplasm via signaling pathways or in the nucleus via interactions with host DNA-binding proteins. Even though there are different reports describing many cell pathways modified by HBx, and its numerous binding–interacting partners, a detailed mechanism that regulates its various functions has been complicated to unify. To date, there are no other published reports where the effects of the phosphorylation of HBx has been systematically correlated with the subcellular location of this viral protein. Since HBx is a pivotal factor for HBV replication, studies of protein regulation are of fundamental interest.

Protein phosphorylation is a well-known regulatory event that plays a key role in the control of multiple functions of viral and cellular proteins [41,42,43]. Modulation of protein functions can affect multiple mechanisms including regulation of subcellular localization, protein–protein interactions, protein stability, and protein-nucleic acid binding, as previously reported for viruses such as varicella-zoster virus [44], human papillomavirus [45], rotavirus [46], rabies virus [47], and hepatitis C virus [48,49], among several others [43]. For the *Hepadnaviridae* family members, protein phosphorylation is a central event during the replication of the DNA genome. The phosphorylation status of HBV nucleocapsids has been shown to reflect the maturation stage of the viral particles [50,51]. Previously, utilizing bioinformatics analysis, our group showed that the conserved residues of the primary sequence of HBx, Ser25, Ser41, and Thr81 exhibited a high potential to be phosphorylated via a conserved mechanism of post-translational modification [32]. In the current study, we thought to validate our previous bioinformatics data. The three-dimensional structure of HBx is unknown. However, it is organized into two functional domains: the N-terminal first third displays a negative regulatory domain (the first fifty amino acid residues), while the two-thirds C-terminal region has a function as a transactivation region [5,6]. As shown in Figure 1A, both Ser25 and Ser41 are located within the Ser/Pro-rich region of the negative regulatory region whereas Thr81 is located within the transactivation C-terminal domain. We hope that the three-dimensional structure of HBx can be solved in the future to elucidate the microenvironment of both Ser41 and Thr81 locations at the sub-protein level.

**Figure 5:**
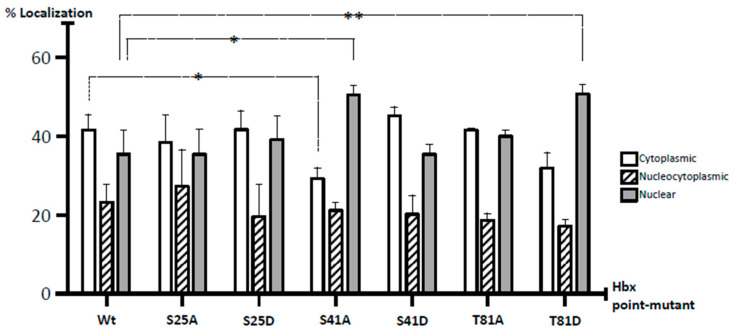
Subcellular distribution of the WT HBx-GFP and HBx-GFP point mutants. The standard deviation was obtained from three independent experiments. * *p* < 0.05, ** *p* < 0.01.

We tested whether the phosphorylation state of each of the conserved amino acid residues Ser25, Ser41, or Thr81 regulated the subcellular localization of the HBx protein. For this, mutational analysis of each of these residues was performed by substituting them for either aspartate or alanine residues. These assays were established by Thorsness et al. [52], demonstrating that the substitution of the phosphorylated Ser for an Asp or Glu residue was able to mimic the phosphorylated state of the enzyme isocitrate dehydrogenase. The reasoning for this statement is that acidic residues are negatively charged such as phosphoserine, phosphothreonine, or phosphotyrosine, even though glutamic acid and aspartic acid only have one negative charge, while phosphoserine, phosphothreonine, and phosphotyrosine are mainly doubly charged at a physiological pH [53]. In contrast, substitution by Ala, an amino acid structurally similar to Ser, mimics a state of absence of phosphorylation by the lack of the nucleophilic group (–OH). Using this methodology, we determined that the subcellular location of the HBx protein can be modulated by simulating either the non-phosphorylation state of Ser41 (S41A) or the phosphorylation state of Thr81 (T81D). In contrast, we also found that Ser25 (either S25A or S25D) has a non-distinguishable behavior from that of the WT HBx protein. Quantitative analysis of WT HBx subcellular distribution indicated that the protein was 41% localized in the cell cytoplasm, 23% was nucleocytoplasmic, and 35% was a nuclear protein (Figure 5). Interestingly, the nucleocytoplasmic distribution of WT HBx and point mutants remained close to approximately 20% as indicated by Figure 5, perhaps indicating a main sorting location.

Since it was previously observed that different expression levels of HBx determine different subcellular distributions, three different amounts of input DNA (low, medium, and high) were used for transfection [24]. By using fluorescence, it was observed that when the WT HBx-GFP protein was expressed at low levels, it displayed a mainly nuclear subcellular location. In the same way, very similar results were obtained for all point mutants in the conserved amino acid residues of HBx Ser25, Ser41, and Thr81, indicating that the mutations did not generate any alteration in the distribution under these assay conditions (at low level of protein expression). In contrast, when increasing the input DNA up to medium or high amounts, WT HBx as well as the point mutants such as S25A, S25D, S41D, and T81A began to be accumulated in the cytoplasm. This indicated that either the nuclear import or nuclear retention capability of HBx would be limited under these conditions. These results are consistent with those obtained by Cha et al. [24] and Henkler et al. [23], when transfecting human hepatoma cells with different amounts of DNA, the accumulation of HBx in the cytoplasm was observed. This phenomenon of accumulation of the HBx protein in the cytoplasm may take place because HBx bears a nuclear export signal (NES) motif that is conserved between the different HBV genotypes [54]. This HBx NES is capable of interacting and sequestering the nuclear export receptor, Crm1 (Chromosomal Maintenance 1, or Exportin 1). This finding was demonstrated through mutational analysis of the HBx NES, indicating that this primary sequence is essential to maintain the cytoplasmic location of the viral protein [54]. Additionally, the association of HBx to Crm1 might suggest the pleiotropic effects of the HBx protein, since Crm1 transports and controls several host factors including NF-κB (nuclear factor kappa-light-chain-enhancer of activated B cells), NFAT (Nuclear factor of activated T-cells), MAPKK (Mitogen-activated protein kinase kinase, also known as MAP2K, MEK, MAPKK), and p53 [55], whose activities are known to be targeted by HBx. Therefore, promiscuous HBx-mediated transactivation might be a secondary effect resulting from impaired Crm1 function. Similar to the HBx protein, other viral trans-activator oncoproteins such as the Rev protein of HIV-1 and Rex of HTLV-1 also possess a NES.

In contrast, opposite results were observed with both HBx mutant proteins S41A and T81D, which both displayed preferential nuclear localization. For both of these HBx point mutants, there was a significant increase in their subcellular distribution, with more than 50% nuclear localization (Figure 5). Thus, contrary to the results of the WT HBx protein and the rest of the tested mutants, both mutant proteins S41A and T81D did not display cytoplasmic accumulation when transfecting the entire range of plasmid DNA amounts (low to high). Therefore, this indicated that the retention in nuclear compartmentalization was due to an effect of the mutation present in each case. Previously, Noh et al. [30] showed that the HBx Ser41 residue was capable of being phosphorylated by the ERK1/2 protein kinase, affecting the HBx subcellular location. However, they described that the S41A substitution in HBx caused the protein to be accumulated in the cytoplasmic compartment. These results are in contrast to those obtained herein, but this might reflect the use of different experimental methodologies. In our case, as previously described, we used a whole range of transfected DNA amounts, obtaining consistent results within a dynamic subcellular distribution of this viral protein. Conversely, it is known that the HBV HBx protein does not bear a nuclear localization signal motif. It is thought that due to the small size (17 kDa) of HBx, the protein would be able to passively diffuse through the nuclear pore complex [54]. Thus, using artificial proteins, the maximum size of a protein to diffuse through the nuclear pore is approximately 60 kDa [56]. Therefore, the effects of individually HBx S41A and T81D point mutants could explain their nuclear retention.

The relevance of HBx Ser41 phosphorylation was also previously studied by Pang et al. [31]. In their report, they were able to determine that Pin1 (a peptidyl-prolyl cis-trans isomerase) bound to Ser41 within the Ser/Pro-rich region of HBx, but the binding was dependent on phosphorylation of the Ser41 residue. This interaction was able to increase the stability of HBx, which correlated with the improvement of transcriptional activity and the development of HBx-induced hepatocellular carcinomas in a model system [31]. Therefore, it might be speculated that the absence of the phosphorylation of HBx Ser41 would affect its binding with Pin1, thus, affecting the oncogenic potential of the viral protein.

To have a further reference of HBx subcellular localization, the co-expression of WT HBx and point mutants with a mitochondrial targeting sequence fused to a red fluorescent protein (DsRed, from *Discosoma* sp.) was analyzed. It was observed that WT HBx as well as some of the HBx point mutant proteins such as S25A, S25D, S41D, and T81A colocalized with the reporter protein signal at the mitochondria. In addition, the association of HBx proteins with the mitochondria generated an abnormal mitochondrial distribution within the cell cytoplasm, which is preferably located in the nuclear periphery. These results are consistent with several previous reports, which have observed HBx protein co-localization with either mitochondria [23,36,57] or mitochondrial proteins [57,58,59]. It was later described that the association of HBx with the mitochondria was due to the presence of a mitochondrial destination sequence in the primary sequence of HBx between residues 111 and 116 [60]. However, surprisingly, the HBx proteins localized in the nucleus such as S41A and T81D also affected some of the mitochondrial distribution within the cell cytoplasm.

The HBV replicative intermediate, the covalently closed circular DNA (cccDNA), is responsible for the persistent infection of hepatocytes. This cccDNA intermediate is the template for the transcription of all viral RNAs, which reflects viral replication [4]. Inside the nucleus, the HBV DNA genome is organized as a mini chromosome, forming the typical “beads on a string” structure of the cellular chromatin [61]. In addition to cellular histones, other proteins help to compact the viral DNA. Unlike cellular chromatin structure and the function of the DNA, little is known about the regulation of infection by HBV chromatin [62]. It has been reported that histones H3 and H4, associated with the HBV mini chromosome, are post-translationally modified [62,63]. Consistently, HBV viral replication is temporally correlated with the acetylation of H3 and H4 mini chromosomes, and with the recruitment of enzymes that acetylates the histones such as p300 and CBP [64,65]. Interestingly, the HBx viral protein interacts with p300, suggesting that the viral protein could regulate the acetylation levels of the HBV minichromosomal histones. It will be highly significant to investigate the role of both nuclear HBx (phospho)S41, and HBx (phospho)T81 when interacting with p300 within a chromatin context, and how this interaction could regulate viral replication.

In contrast, it has been proposed that HBx does not directly bind to DNA sequences but through other factors. However, using chromatin immunoprecipitation (ChIP)-based quantitative assays, it was shown that HBx was recruited to the cccDNA mini chromosome, and the kinetics of HBx recruitment onto the cccDNA paralleled HBV replication. Moreover, p300 recruitment was severely impaired, and cccDNA-bound histones were rapidly hypoacetylated in cells replicating the HBV HBx-deficient mutant, whereas the recruitment of the histone deacetylases hSirt1 and HDAC1 was increased and occurred at earlier times [64,66]. It has recently been proposed that HBV transcription from cccDNA is repressed in the absence of HBx, and this repression is characterized by a compacted structure of the cccDNA. Currently, a model of HBV transcriptional activation has been proposed in which HBx recruitment to the HBV mini chromosome serves to counteract chromatin-mediated transcriptional repression. In the model, there would be cellular factors inducing silencing in the absence of HBx, but in its presence, HBx counteracts their activity [62]. Since in the current regulation model the HBx protein displays an enhancing activity, it will be highly interesting to define the function of nuclear HBx phosphorylation, especially in the search for new potential targets for antiviral blocking.

It is known that HBx became phosphorylated when expressed in insect cells and human hepatoma cell HepG2 [26,27]. Moreover, recombinant HBx was found to be in vitro phosphorylated by protein kinases. Phospho-amino acid analysis later showed that the phosphorylated residues of the protein could correspond to Ser residues [28]. It has also been determined that Ser residues at position 31 and 41 are phosphorylated by protein kinases Akt 1 and ERK1/2, respectively [29,30]. Applying bioinformatics tools to investigate whether the HBx protein might be regulated via phosphorylation, our group previously found that the phylogenetically conserved residues Ser25 and Ser41, and Thr81 are highly predicted to be phosphorylated [32]. In the current study, and to validate our prediction data, phylogenetically conserved amino acids of HBx with a high potential of phosphorylation were targeted for site-directed mutagenesis. Overall, we positively validated our own previous bioinformatics study and we found that within cells, there were different populations of the HBx protein, where some of them were cytoplasmic some others were nuclear proteins. These different HBx populations are possibly regulated by phosphorylation amongst other regulatory mechanisms at diverse subcellular locations.

## 4. Materials and Methods

### 4.1. Site-Directed Mutagenesis of Phosphorylation Point Mutants of the HBx Protein

We have recently reported the cloning of a full HBV functional genome (Clone 4.5, genotype F1B from Chile) from the serum of a chronic infected Chilean patient [33,34]. The wild-type sequence of HBV HBx from Clone 4.5 was individually moved into a cloning plasmid (pCR-XL-TOPO, Invitrogen), and the sequence was confirmed by DNA sequencing. For mutagenesis of HBx, we selected the positions Ser25 and Ser41 (both within the negative regulatory domain), and Thr81 (in the transactivation domain), and these residues were point mutated to either aspartic acid (to mimic a constitutive phosphorylation; S25D, S41D, and T81D) or alanine (to mimic an amino acid that cannot be phosphorylated; S25A, S41A, and T81A). The cloning plasmid containing the wild-type sequence of HBx was incubated with a pair of primers (Table S1), and site-directed mutagenesis was performed with the QuikChange II Kit (Agilent Technologies). Sequences of selected clones were confirmed by sequencing the two strands of the plasmid DNA. Corresponding DNA fragments containing either HBx wild-type or phosphorylation point mutant sequences were amplified by PCR using a pair of primers, as indicated in Table S2, to make constructs for final cloning into expression vectors, either pcDNA3.1-3xFLAG (Invitrogen) or pAcGFP1-N1 (Clontech). First, in the case of wild-type and point mutants in the expression vector pcDNA3.1-3xFLAG, viral proteins were expressed as a fusion polypeptide containing the 3xFLAG tag at the N-terminus of HBx. In the case of wild-type and point mutants in the expression vector pAcGFP1-N1, the viral proteins were expressed as a fusion polypeptide containing the GFP tag (reporter protein) at the C-terminus of HBx.

### 4.2. Cells, Cell Culture, and Transfections

The human hepatocarcinoma Huh-7 and HepG2 cell lines were grown in Dulbecco’s modified Eagle’s medium (DMEM), supplemented with 10% fetal bovine serum (FBS), 100 U/mL penicillin, 100 ug/mL streptomycin, and 2 mM glutamine. The cells were incubated at 37 °C and 5% CO_2_. To harvest the cells, they were trypsinized and collected in completed DMEM. Cells were seeded no more than 24-h before the start of the experiment.

All transient transfections on either Huh-7 or HepG2 cell lines were carried out using Lipofectamine 2000 (Invitrogen, Life Technologies, Carlsbad, CA, USA), with a ratio of DNA (µg): Lipofectamine (µL) = 1:2.5. Three different amounts of plasmid DNA were utilized, corresponding to low (0,64 µg), medium (1,6 µg), or high (4 µg) for a standard 100 mm tissue culture dish, and cells at 70% confluency at the transfection. All transient transfection reactions were carried out with Opti-MEM (Gibco, Life Technologies) media. At 6-h post-transfection, the media was changed and replaced by complete media until cells were processed. We also carried out co-transfection experiments to overexpress HBx-GFP protein and point mutants along with a plasmid vector (pDsRed2-Mito) encoding a mitochondrial targeting sequence fused to a red fluorescent protein, DsRed (from *Discosoma* sp., Clontech, Mountain View, CA, USA) [38,39].

### 4.3. Western Blots

To analyze the peak abundance of either HBx-GFP or 3xFLAG-HBx proteins, we carried out a time-course experiment. For this, plasmids containing each HBx construct were transfected, and cells were lysed for analyses 6-, 24-, 48-, and 72-h post-transfection. For the analyses of the expression of HBx and point mutant proteins, either pcDNA-3xFLAG or pAcGFP plasmids containing the corresponding mutated sequences were transfected, and 24-h post-transfection, cells were lysed for analyses. Washed cell pellets were resuspended with RIPA buffer (Radioimmunoprecipitation assay buffer, 150 mM NaCl, 1% NP-40, 0.1% SDS, 50 mM Tris pH 8.0, protease inhibitor cocktail (Roche, Basel, Switzerland), insoluble cell debris were discarded by centrifugation, and total proteins were quantified by the Bradford method (Quick Start TM Bradford, BioRad, Hercules, CA, USA). Fifty µg of total proteins for each sample were resolved by 12.5% SDS-PAGE (Sodium dodecyl (lauryl) sulfate-polyacrylamide gel electrophoresis), and then transferred to nitrocellulose membranes that were probed with the indicated antibodies, and reactions were developed by chemiluminescence (Pierce ECL Western blotting, Thermo Scientific, Waltham, MA, USA) and exposed to autoradiography films (Orto CPG-GU, Agfa, Mortsel, Belgium).

### 4.4. Indirect Immunofluorescence

Approximately, 3.5 × 10^5^ cells were seeded on 12 mm coverslips, and 24-h later, cells were transfected with plasmids containing HBx constructs such as pcDNA-3xFLAG-HBx wild-type and phosphorylation mutants. Cells were washed three times with 1 × Phosphate-buffered saline (PBS) 24-h post-transfection and fixed with 4% paraformaldehyde (PFA) in PBS for 10 min at room temperature. Cells were then washed three times for 5 min with 1 × PBS, and permeabilized with 0.5% Triton X-100 in PBS for 15 min. Cells were washed with 1 × PBS, and blocked with 10% BSA in 1 × PBS and incubated at 37 °C for 30 min in a humidity chamber. Cells were incubated with mouse monoclonal antibody anti-FLAG M2 (Sigma Aldrich (F1804), St. Louis, MO, USA), dilution 1:700) in blocking solution (3% BSA in 1 × PBS) for 1 h at 37 °C in a humidity chamber, continuing with three washes of 3 min each with 1 × PBS. Cells were then incubated with chicken secondary antibodies, anti-mouse conjugated with Alexa 488 (Life Technologies (A-21200)) in blocking solution (3% BSA in 1 × PBS) for 45 min at 37 °C in a humidity chamber. Cells were then washed three times with 1 × PBS, and nuclei were counterstained with 0.2 µg/mL DAPI in 1 × PBS, and washed for 5 min with water. Treated coverslips were mounted on slides with 5 µL of Fluoromount-G (Southern Biotech Inc., Birmingham, AL, USA), and dried at room temperature. Cells were visualized on an Olympus FSX Bio Imaging Navigator.

### 4.5. HBx-GFP Epifluorescence

Approximately 3.5 × 10^5^ cells were seeded on 12 mm coverslips, and 24 h later, cells were transfected with plasmid containing HBx constructs such as pAcGFP-HBx wild-type and phosphorylation mutants. Cells were washed three times with 1 × PBS 24-h post-transfection and fixed with 4% PFA in 1 × PBS for 10 min at room temperature. Cells were then washed three times for 5 min each with 1 × PBS, and then the nuclei were counterstained with 0.2 µg/mL DAPI in 1 × PBS, and then washed for 5 min with water.

### 4.6. Quantitative Analysis of the Subcellular Localization of the HBx-GFP Protein and Point Mutants

A total of approximately 200 positive cells for the expression of GFP of either WT or point mutant HBx proteins were analyzed from three different experiments. Cells were transfected with one fixed amount of DNA (high amount) for WT HBx-GFP as well as point mutants, and after 24-h, coverslips were processed for fluorescence. Expression of HBx-GFP proteins was carefully associated to either the cytoplasm, nucleus, or nucleocytoplasmic compartments with respect to DAPI-positive nuclear staining. The standard deviation was obtained from three independent experiments. * *p* < 0.05, ** *p* < 0.01, *** *p* < 0.001.

## Data Availability

The datasets used and/or analyzed during the current study are available from the corresponding authors on reasonable request.

## Supplementary Materials

The following are available online, Table S1: PCR primers used for site directed mutagenesis; Table S2: PCR primers used for cloning in expression vectors.

## Sample Availability

Samples of the compounds are available from the authors.
