# Supplementary material for: Phosphorylation of Phylogenetically Conserved Amino Acid Residues Confines HBx within Different Cell Compartments of Human Hepatocarcinoma Cells
Source: Molecules. 2021 Feb 26;26(5):1254. doi: 10.3390/molecules26051254 (PMC7956559; doi:10.3390/molecules26051254)
Supplement: Supplementary file 1 [file molecules-26-01254-s001.pdf]

Table S1. PCR primers used for site directed mutagenesis

Name	Construct	Direction	5´-3´ Sequence[a]
HBx-S25A	S25A	Forward	5´-TCCCGTCAGCGCTGAA**G**CCTGCGGACGATCCCTCTCG-3´
HBx-S25A	S25A	Reverse	5´-CGAGAGGGATCGTCCGCAGG**C**TTCAGCGCTGACGGGA-3´
HBx-S25D	S25D	Forward	5´-TCCCGTCAGCGCTGAA**GA**CTGCGGACGATCCCTCTCG -3´
HBx-S25D	S25D	Reverse	5´-CGAGAGGGATCGTCCGCAG**TC**TTCAGCGCTGACGGGA-3´
HBx-S41A	S41A	Forward	5´-GGGCTGTATCGCCCCCT**G**C**G**CCGTCTGCCGTTCCAGC-3´
HBx-S41A	S41A	Reverse	5´-GCTGGAACGGCAGACGG**C**G**C**AGGGGGCGATACAGCCC-3´
HBx-S41D	S41D	Forward	5´-GGGCTGTATCGCCCCCT**GA**TCCGTCTGCCGTTCCAGC-3´
HBx-S41D	S41D	Reverse	5´-GCTGGAACGGCAGACGGA**TC**AGGGGGCGATACAGCCC-3´
HBx-T81A	T81A	Forward	5´-TCTGCACTCGCATGGAG**G**CCACCGTGACCGCCCCTCG-3´
HBx-T81A	T81A	Reverse	5´-CGAGGGGCGTTCACGGTGG**C**CTCCATGCGACGTGCAGA-3´
HBx-T81D	T81D	Forward	5´-TCTGCACGTCGCATGGAG**GA**CACCGTGAACGCCCCTCG-3´
HBx-T81D	T81D	Reverse	5´-CGAGGGGCGTTCACGGTG**TC**CTCCATCGACGTGCAGA -3´

[a] Bold and underlined, mutagenized nucleotide(s).

Table S2. PCR primers used for cloning in expression vectors

**Construct** (Vector)	**Direction**	**5´-3´ Sequence[a]**	**Restriction Site**
3xFLAG-HBx (pcDNA3.1-3XFLAG)	Forward	5´-GATCGATCGATC**AAGCTT**ATGGCTGCTCGGTTGTGCTG-3´	HindIII
	Reverse	5´-GATCGATCGATC**GAATTC**TTAGGCAGAGGTGAAAAAGTTG-3´	EcoRI
HBx-GFP (pAcGFP)	Forward	5´-GATCGATCGATC**AAGCTT**ATGGCTGCTCGGTTGTGCTG-3´	HindIII
	Reverse	5´-GATCGATCGATCC**GGTACC**GTGGCAGAGGTGAAAAAGTTGCA-3´	KpnI

[a] Bold and underlined, restriction site.
